# The Cost of Health Care Services in Urban and Intercity Road Traffic Accidents

**Published:** 2019-09

**Authors:** Alireza AMANOLLAHI, Mohammad HOSEINI KASNAVIEH, Nader TAVAKOLI, Mohammad VEYSI, Ali TAHMASEBI

**Affiliations:** 1. Trauma and Injury Research Center, Iran University of Medical Sciences, Tehran, Iran; 2. Health Management and Economics Research Center, Iran University of Medical Sciences, Tehran, Iran

## Dear Editor-in-Chief

This cross-sectional analytical study was conducted from Apr to Sep 2017 on 2933 people injured in west Tehran’s inner-city (811; 27.6%) and south of the intercity (2122; 72.4%) Road Traffic Injuries (RTIs). Ethical code of this study was approved in Iran University of Medical Sciences.

The injured treated as outpatients and those who stayed in the hospital less than six hours were excluded, and only the patients who received medical services as inpatients were included in the study. The cost of medical services, the length of hospital stays and the factors affecting them were compared between the two hospitals. The distribution of data was abnormal. Multi regression analyses were done after data transformation (log) and normalization.

Patients’ Length of Stay (LOS) differed between two groups. (Trauma center 8.7 vs. general hospital 7.2 *P*=0.03). The median of the total costs of medical services provided to the hospitalized patients (Table 1) was 3915 $ in the trauma center and 9167 $ in the general hospital. Mann-Whitney’s U-test showed the costs incurred in these two hospitals (*P*<0.001).

**Table 1:** Cost of health services provided by type of hospital

***Bed and services***	***General Hospital***	***811***	***Median: 2054.7*** ***IQR= 4211.7***	***< 0.001***
	Trauma center	2122	Median: 1182.9 IQR= 1963.3	
Operating room and orthopedic equipmen	General Hospital	402	Median: 125.3 IQR= 203.3	< 0.004
	Trauma center	2018	Median: 825.9 IQR= 924.2	
Surgeon’s fees	General Hospital	402	Median: 564 IQR= 917.5	< 0.04
	Trauma center	1789	Median: 393.5 IQR= 392.2	
Medications	General Hospital	774	Median: 944.6 IQR= 1146.6	< 0.02
	Trauma center	2112	Median: 2054.7 IQR= 4211.7	
Radiology and sonography	General Hospital	783	Median: 975.2 IQR= 1263.2	< 0.001
	Trauma center	1830	Median: 103.8 IQR= 99.5	
Anesthesia	General Hospital	364	Median: 283.1 IQR= 274.5	< 0.001
	Trauma center	1789	Median: 133.8 IQR= 126.7	
CT Scan	General Hospital	631	Median: 196 IQR= 176.9	< 0.06
	Trauma center	1343	Median: 144.5 IQR= 150.1	
Physiotherapy	General Hospital	51	Median: 29.8 IQR= 70.3	< 0.001
	Trauma center	792	Median: 90.2 IQR= 238.5	
Procedures	General Hospital	677	Median: 60.8 IQR= 93.6	< 0.001
	Trauma center	1764	Median: 108.9 IQR= 124.8	
ECG	General Hospital	280	Median: 17 IQR= 11.5	< 0.001
	Trauma center	470	Median: 2.5 IQR= 4.8	
Total Cost	General Hospital	811	Median: 9167.4 IQR= 10701.1	< 0.001
	Trauma center	2122	Median: 3915 IQR= 4550	

Table 2 presents the beta coefficients for the significant variables of the multiple linear regression. In the regression modeling for determining the factors affecting the total hospital costs, the variables included in the model estimated 82% of the costs, and the costs of physiotherapy, radiology and sonography, operating room and orthopedic equipment, surgeon’s fees, LOS and bed and services had the highest impact.

**Table 2:** Efficiency of the total cost based on the cost of services received

***Model***	***β***	***T***	***P-value***
(Constant)	1.91	3.42	.000
Physiotherapy	.102	2.47	.015
Radiology and sonography	.105	2.36	.02
Equipment of operation and orthopedic	.206	3.35	.001
Surgery	.161	2.56	.01
Drug	.15	1.95	.053
Bed and Services	.17	2.06	.041
LOS	.166	2.48	.014

The cost of treatment incurred by patients was 34,160 Euros in slight accidents and 121,925 Euros in serious accidents; ultimately, the cost of the accident was proportional to the number of injured limbs (1). Mean cost of serious accidents was four times higher than the cost of medium accidents and 26 times higher than that of slight accidents, and the cost of medium accidents was also six times higher than that of slight accidents (2).

The total cost in this study of the injured in RTIs was higher in the general hospital compared to the trauma center due to providing a variety of medical services and subsequently, the complexity of the hospital procedures, the role of other costs was more highlighted in the general hospital. Therefore, cost managing is an essential matter in general hospitals for avoiding inappropriate services to patients.

Shorter trauma and admission time in specialized trauma centers compared to community hospitals (3). The transfer of RTI patients to trauma centers shortens the LOS and also decreases costs; however, the cost was higher in the general hospital than the trauma center.

LOS and hospital costs were significantly higher in RTI patients with nosocomial infections compared to the patients without such infections (4). Undergoing surgery, being male, being old and dealing with a range of damages are associated with a significantly longer LOS and higher mortality rate, while LOS >6 d was associated with higher mortality. The Revised Trauma Score and Injury Severity Score differed between the dead and alive patients (4, 5). The heavy costs and socioeconomic implications of RTIs clearly require special attention from policy-makers for preventive measures (6).
